# Biological Activity and Potential Health Benefits of Edible *Prunus* Fruits: A Narrative Review

**DOI:** 10.3390/plants15121891

**Published:** 2026-06-18

**Authors:** Piotr Służały, Irma Podolak, Agnieszka Galanty

**Affiliations:** 1 Doctoral School of Medical and Health Sciences, Jagiellonian University Medical College, 16 Łazarza Str., 31-530 Kraków, Poland; piotr.sluzaly@doctoral.uj.edu.pl; 2 Department of Pharmacognosy, Jagiellonian University Medical College, Medyczna 9, 30-688 Kraków, Poland; irma.podolak@uj.edu.pl

**Keywords:** *Prunus*, biological activity, Rosaceae, chemoprevention, cytotoxic, antioxidant, anti-inflammatory

## Abstract

This review aims to compare the biological properties of eleven fruits of the *Prunus* species, with the focus on their potential in the prevention and management of chronic diseases. The search spanned publications from 2000 to May 2026, only in English, and utilized databases such as PubMed, Web of Science, and Google Scholar, focusing on the in vitro and in vivo studies. The exclusion criteria included review articles, studies focusing exclusively on isolated phytochemicals or synthetic derivatives from *Prunus* species, and in silico or theoretical analyses. The fruits of *Prunus* species exhibited a broad spectrum of activities, including antioxidant, anti-inflammatory, anticancer, antihyperglycemic, or neuroprotective. Interestingly, sour cherries exhibited sleep-enhancing, and xanthine oxidase-inhibitory effects, while apricots showed promising hepatoprotective activity. Key species, including apricots, cherries, peaches, and plums, are widely recognized for their bioactive phytochemicals and potential health benefits, while some (e.g., bird cherry, blackthorn) are less examined, although promising. *Prunus* fruits revealed health-benefit potential, that at least partially supports their ethnopharmacological uses. However, further clinical and mechanistic studies are warranted to validate their efficacy and explore potential applications in pharmaceutical formulations.

## 1. Introduction

The genus *Prunus* L., part of the Rosaceae family, includes approximately 430 species categorized into five subgenera: *Padus*, *Amygdalus*, *Cerasus*, *Prunophora*, and *Laurocerasus*. These species consist of both deciduous and evergreen trees and shrubs, predominantly found in the temperate regions of the Northern Hemisphere. Many of these species hold significant horticultural value, with several being cultivated for their edible seeds and fruits. Notable examples include plums, peaches, cherries, and apricots [1]. While most species within this genus are primarily grown for their fruits, some are also utilized for ornamental purposes or timber production. Members of the genus produce fleshy drupe fruits, including economically important species such as plums, peaches, cherries, and apricots. Unlike almonds, where the seed is consumed, most stone fruits have their edible portion consisting of the mesocarp and/or exocarp. *Prunus* species are highly valued as temperate fruits, appreciated by consumers for their taste, color, and sweetness. They are also the subject of research for their nutritional and bioactive properties, which could be beneficial to human health [2,3], with still increasing scientific interest. Despite a number of different review articles, usually focusing on phytochemistry and pharmacological activity of a single *Prunus* species [4,5], so far there is no work which compares the possible health benefits of different edible *Prunus* fruits. Thus, the review aims to summarize the current knowledge on biological properties of the fruits of the edible *Prunus* species that can possibly provide some benefits for human health.

## 2. Search Strategy

**Figure 1:**
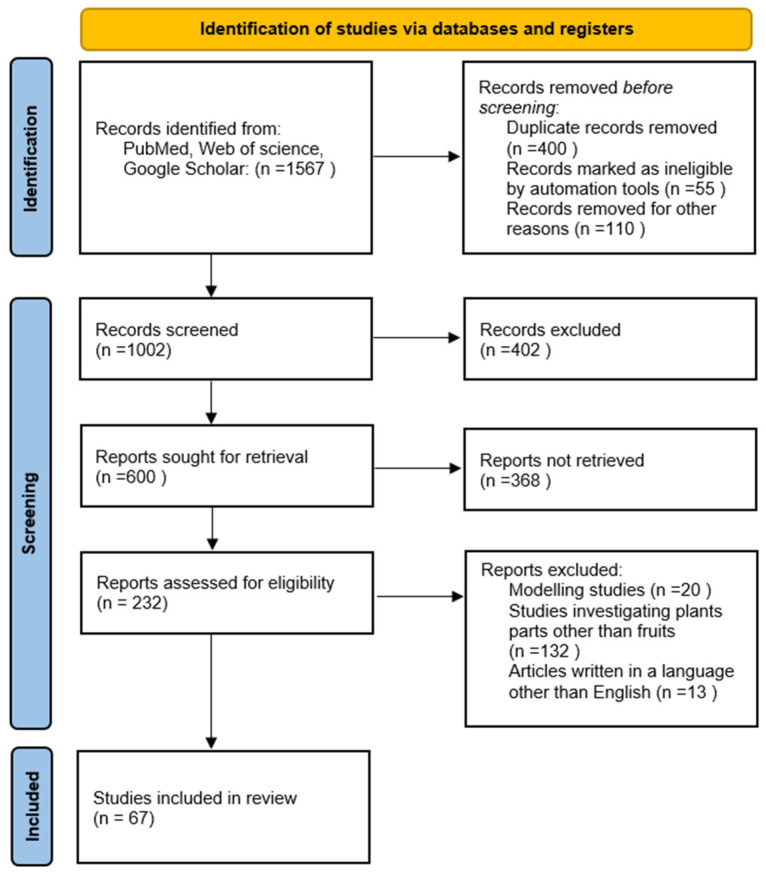
Search strategy diagram for the studies included in the review.

A comprehensive examination was conducted on the existing literature and published research, documenting in vitro and in vivo experimental studies, as well as clinical studies (Figure 1). The focus was on the cytotoxic, anticancer, antioxidant, anti-inflammatory, and other biological effects associated with *Prunus* species. The selection of *Prunus* species was predefined prior to the literature review and based on the following inclusion criteria: edible fruit use, documented ethnomedicinal relevance, and availability of peer-reviewed studies describing phytochemical composition and biological activities (antioxidant, anti-inflammatory, cytotoxic effects). Species lacking sufficient experimental evidence or biomedical relevance were excluded during the screening process. The literature search spanned publications only in English from 2000 to May 2026, and utilized databases such as PubMed, Web of Science, and Google Scholar. Key terms employed in this search included: “Prunus”, including the particular species “*Prunus armeniaca* L.”, “*Prunus avium* (L.) L.”, “*Prunus cerasoides* Buch.-Ham. ex D. Don”, “*Prunus cerasus* L.”, “*Prunus domestica* L.,” “*Prunus mahaleb* L.,” “*Prunus padus* L.” “*Prunus persica* (L.) Batsch”, “*Prunus serrulata* Lindl.”, “*Prunus spinosa* L.”, “*Prunus serotina* Ehrh.”, and also their common English names (e.g., plum, cherry, bird cherry, apricot, blackthorn), combined with “anticancer”, “antioxidant”, “anti-inflammatory”, “cytotoxic”, “antidiabetic”, “antimetabolic”, “hepatoprotective”, “urinary”, “neuroprotective”, or just “activity”. The exclusion criteria encompassed review articles, studies not available in full text or not published in English, and the investigations focusing exclusively on isolated phytochemicals or synthetic derivatives from *Prunus* species. Additionally, purely in silico or theoretical analyses, as well as those unrelated to medicinal or pharmacological applications of *Prunus* species, were also excluded.

## 3. Traditional Uses

The genus *Prunus* encompasses a wide variety of species that have been extensively utilized in traditional medicine systems across Asia, the Middle East, and other regions. These species, including *P. persica*, *P. armeniaca*, *P. cerasus*, and others, are valued for their diverse pharmacological properties and are incorporated into therapeutic formulations in systems such as Unani, Traditional Chinese Medicine (TCM), Ayurveda, and Iranian Traditional Medicine. Across these systems, different parts of *Prunus* plants, including fruits, are employed in the treatment of various ailments, ranging from respiratory and gastrointestinal disorders to neurological and hepatic conditions [6,7]. *Prunus avium* fruits are available for purchase from herbal dispensaries in Iran to prepare decoction, indicated for the relief of renal stones, edema, and hypertension [8]. The fruits of *Prunus cerasoides* have been utilized in Himalayan traditional medicine for its therapeutic properties, particularly in the treatment of wound healing, backaches, and sprains [9]. *Prunus cerasus* is recognized in traditional Indian medicine for its various therapeutic properties. The fruit is believed to possess stomachic, purgative, and tonic effects, particularly beneficial for the brain [10]. *Prunus domestica* fruits have been traditionally utilized in India for its medicinal properties, including its roles as a laxative, refrigerant, appetite stimulator, stomachic, digestive aid, and tonic. It is commonly employed to address conditions associated with an imbalance of Pitta, as well as symptoms such as nausea, flatulence, colic, dyspepsia, and general debility [11]. The fruit of *P. persica* is used in India as a demulcent, an antiscorbutic, and a stomachic [12]. In Mexico, *Prunus serotina* has been recognized for its nutritional and medicinal applications. The fruit is frequently enjoyed in its fresh form, as well as in the preparation of jams, liquors, and syrups. The fruits are also utilized in the treatment of diarrhea and cough [13,14]. The fruits of *Prunus padus* continue to be utilized as anesthetics and disinfectants in Russia and various Scandinavian countries. Furthermore, the fruits have demonstrated efficacy in addressing dysentery, digestive issues, and stomach discomfort. In Eastern and Northern Europe, it is customary for individuals to dry, crush, and mill the fruits, incorporating them into tart fillings or combining them with flour for baking purposes. In Russia, bird cherry liqueur and concentrated juices are produced from the fruits, imparting a deep red hue to the liquids [15].

## 4. Biological Activity

### 4.1. The Issue of Extract Standardization

The biological activity of plant-derived extracts is strongly influenced by multiple experimental and biological factors, including extraction methodology, solvent polarity, cultivar variability, assay conditions, and the maturity stage of the fruits. Consequently, the interpretation and comparison of bioactivity data should be approached with considerable caution. Although numerous in vitro studies have demonstrated promising antioxidant and other pharmacological properties, further in vivo and human studies are required to confirm their physiological relevance and clinical significance.

A representative example is provided by one of the studies [8], conducted on fresh and dried fruits of *Prunus domestica*, in which the ethanol and methanol extracts were evaluated for their phytochemical composition, particularly total phenolic and flavonoid content. The authors reported that dried fruits exhibited significantly higher antioxidant activity than fresh samples. This effect was attributed primarily to reduced moisture content and the formation of non-enzymatic reaction products during the drying process, which may contribute to enhanced antioxidant potential. Furthermore, methanolic extracts demonstrated greater extraction efficiency and antioxidant activity compared with ethanolic extracts, likely due to the higher polarity of methanol and its improved ability to solubilize phenolic compounds.

These findings highlight the critical importance of methodological parameters in determining the observed biological activity of plant materials. Even minor variations in extraction procedures, solvent selection, or sample processing may substantially alter phytochemical yield and, consequently, biological efficacy. Therefore, standardized methodologies are essential for obtaining reproducible and scientifically comparable results.

### 4.2. Cytotoxic and Anticancer Activity

**Figure 2:**
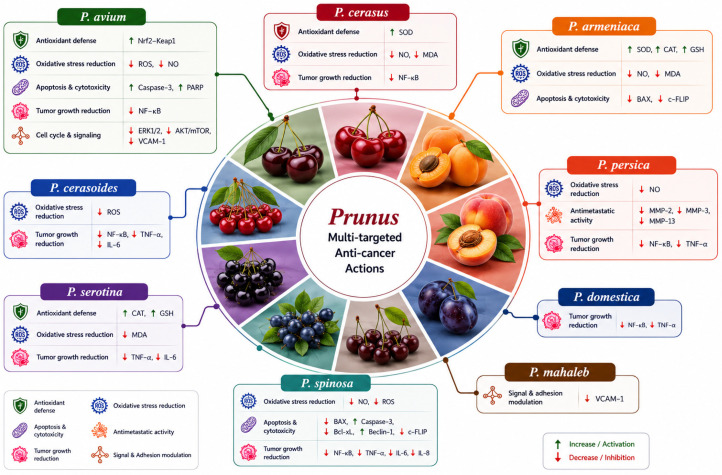
Graphical summary of cytotoxic mechanisms of *Prunus* fruits.

Cytotoxic properties of *Prunus* species in a wide range of cell lines of different origin were described. The details of the reviewed experiments are presented in Table 1, while some interesting results, together with the animal studies, are discussed below. A summary of the cytotoxic mechanisms is presented in Figure 2. Interestingly, no data exists on the cytotoxic effect of fruits of *Prunus cerasoides* and *P. padus*. Only two studies reported the anticancer effect in vivo, as noted for *Prunus cerasus* and *P. persica*.

The extract from *Prunus armeniaca* fruits decreased the proliferation of human breast cancer MCF-7 and MDA-MB-231 cells. However, no phytochemical analysis of the extract was provided, which limits the interpretation of the results [16]. Similarly, *Prunus avium* fruits’ extracts revealed cytotoxic potential to human breast cancer cells, and in one case the effect on colon adenocarcinoma cells was also described. The sweet cherry fruit paste was extracted using an ethanol–dichloromethane mixture under reflux for 5 min. The most interesting effect was noted for ethanol and ethyl acetate extracts from *P. avium* fruits to breast cancer MCF7 cells, with IC_50_ 2.4 and 2.9 µg/mL, respectively [17]. Moreover, some studies reported on the mechanism of the observed cytotoxic effect. Rabelo et al. conducted a study on the effects of total polyphenols and anthocyanins fractions of dark sweet cherries on metastatic 4T1 breast cancer cells. The research demonstrated that polyphenol fraction had an inhibitory effect on reactive oxygen species (ROS), with an IC_50_ value of 58.6 µg/mL. Additionally, the anthocyanin fraction triggered p38-mediated intrinsic apoptosis, resulting in the cleavage of caspase-3 and a decrease in total PARP levels. The study also found a suppression of the ERK1/2 and Akt/mTOR signaling pathways, which are often dysregulated in breast cancer and contribute to increased motility and invasion. This was further corroborated by a reduction in VCAM-1 mRNA expression, decreased Scr phosphorylation, and an 88.6% reduction in cell migration to the wounded area [18]. Another interesting study indicated a synergistic inhibition of cell viability when combining the anthocyanin fraction from dark sweet cherries with doxorubicin, with an IC_50_ value for anthocyanins 244.60 ± 31.62 μg/mL, expressed as cyanidin-3-O-rutinoside equivalents, and 2.51 ± 0.67 μg/mL for doxorubicin. Furthermore, the anthocyanin fraction modulated phase I drug-metabolizing enzymes, leading to a reduction in the activity of cytochrome P450 enzymes that are typically induced by doxorubicin. Additionally, the dosing of doxorubicin was shown to enhance its efficacy while potentially minimizing side effects [19].

Only one study reported on the cytotoxic potential of *P. cerasus* methanolic fruit extract. The main compounds identified in the standardized extract were chlorogenic acid, rutin, quercetin, kaempferol, isorhamnetin, genistein, daidzin, formononetin, catechin, p-coumaric acid, and gallic acid, as characterized by LC-ESI-Q-TOF-MS analysis. The study included five human cancer cell lines of different origin: leukemia THP-1, prostate PC-3, lung A549 and NCI-H322, and breast MCF-7, using MTT and SRB assays. The extract, at a concentration of 50 µg/mL, demonstrated growth inhibition ranging from 40% to 100% in the various cancer cell lines, achieving maximum growth inhibition (100%) in the NCI-H322 cell line, with IC_50_ values of 5.59 and 5 µg/mL as determined by SRB and MTT assays, respectively. Further investigation into apoptosis induction in NCI-H322 cancer cells revealed an increase in the population of apoptotic cells, as evidenced by nuclear morphology studies. The study was further extended to animals, where intraperitoneal administration of the extract at a dosage of 200 mg/kg body weight resulted in tumor growth inhibition of 72.31% in the Ehrlich Ascites Carcinoma (EAC) model and 68.69% in the methylcholanthrene-induced ascites (Meth-A) model in mice [20].

Alsolmei et al. [21] conducted a study on the effects of polyphenol-enriched plum (*P. domestica*) extract, highlighting its ability to enhance myotubule formation and promote anabolism, while also alleviating cellular damage induced by colon cancer in myoblast C2C12 cells. Standardization of the extract showed content of free gallic acid, 3-cholorogenic acid, rutin, free quercetin, and proanthocyanidins. The treatment with plum extract resulted in an almost threefold increase in myocyte cell size and approximately twofold stimulation of myoblast differentiation. Additionally, plum extract promoted total protein synthesis by around 50%, decreased total protein degradation due to serum deprivation by roughly 30%, and led to a twofold increase in the expression of insulin-like growth factor-1 (IGF-1). The study also found that the extract reduced tumor necrosis factor α (TNFα)-induced nuclear factor κB (NFκB) activation by 80% in A549/NF-κB-luc cells. Furthermore, the extract inhibited the growth of Colon-26 cancer cells and mitigated cytotoxicity in C2C12 myoblasts caused by soluble factors released from Colon-26 cells. Navarro et al., in their study on *Prunus domestica* fruits, reported a significant negative correlation between total polyphenolic content and also procyanidins content and cytotoxicity against AGS and SW620 cell lines. The extract was standardized using UPLC-DAD-ESI-MS, where ripe fruits were extracted with 70:30 (*v*/*v*) acetonitrile–water at 40 °C, and the resulting extract was finally processed into a dry powder for analysis, leading to the identification of 52 compounds, including proanthocyanidins (flavan-3-ol monomers such as (+)-catechin and (−)-epicatechin, procyanidin B- and A-type dimers, trimers, tetramers, pentamers, and flavan-3-ol gallates), flavonoids (kaempferol, quercetin, and naringenin derivatives), phenolic acids (protocatechuic, caffeoylquinic, and hydroxycinnamic acid derivatives), hydroxychalcones (phloretin and 3-hydroxyphloretin derivatives), and isoprenoid glycosides (vomifoliol derivatives) [22].

A few studies described the cytotoxic potential of *P. persica*, directed mainly to breast cancer cells. One of them concerned the effects of the fruits on tumor growth, and metastasis of MDA-MB-435 breast cancer cells using in vivo xenograft models. The results indicated significant inhibition of tumor growth and lung metastasis within a dosage range of 0.8 to 1.6 mg/day. These effects appear to be mediated by the suppression of metalloproteinases gene expression. Specifically, the modulation of gene expression for matrix metalloproteinases 2, 3, and 13 may represent key molecular targets for the anti-metastatic activity attributed to peach polyphenolics. The extract contained phenolic acids, anthocyanins, flavonols, and procyanidins (26.7%, 18.5%, 29.3%, and 25.5%, respectively, using chlorogenic acid, cyanidin-3-O-glucoside, quercetin, and catechin as standards) [23].

A number of reports concern the cytotoxic potential of *P. spinosa*, comprising mainly the gastrointestinal (colon, liver) cancer cells, but also studies on glioblastoma, leukemia, prostate and cervical cancer cells. The observed effects were rather moderate or low, with IC_50_ from 55 to 770 μg/mL. Two interesting studies were performed on the combination of *P. spinosa* extract and nutraceutical activator. The first one investigated the cytotoxic activity of Trigno M, an extract derived from *Prunus spinosa* drupes, in conjunction with a nutraceutical activator complex, in 2D and 3D in vitro models, as well as in vivo models, specifically targeting the HCT116 colorectal cancer cell line. The findings demonstrated a noteworthy increase in the apoptotic cell fraction (44.8%) following Trigno M treatment, alongside a minimal necrotic fraction of 6.7% in comparison to control cells. The extract was standardized for its major polyphenolic groups, showing the presence of phenolic acids (39.95 mg/100 g), flavones/flavonols (66.91 mg/100 g), and anthocyanins (100.81 μg/100 g). Furthermore, the extract and the chemotherapeutic 5-fluorouracil induced apoptosis at comparable rates. Additional results indicated a significant delay in the growth of colorectal cancer cells treated with Trigno M relative to the control group [24]. In their subsequent work, the authors examined the effects of a combined treatment of *Prunus spinosa* extract and a nutraceutical activator complex (PsT + NAC^®^) alongside 5-fluorouracil on a three-dimensional model of human colorectal cancer. The findings indicated that this novel combined treatment significantly inhibits autophagy while enhancing apoptotic activity [25].

**Table 1:** Cytotoxic activity of the fruits of *Prunus* species.

Species	Cell Lines	Extract Details	Cytotoxic Activity and/or IC_50_	Reference
*P. armeniaca*	MCF7 and MDA-MB-231	ethanolic fruit extract	significant reduction in cell proliferation	[16]
	A549	ethanolic extract TPC: 662.480 ± 1.931 mg (GA Equi/g) and TPC: 361.660 ± 1.168 (mg QE Equi/g)	significant inhibition of proliferation at 100 and 200 μg/mL	[26]
*P. avium*	Caco-2	ethanolic extract of fruit	IC_50_ 667.84 µg/mL	[27]
	4T1 TNBC	↑ Nrf2-Keap1 activation, impaired autophagy; no GST/GGT antioxidant-cytotoxic response at low dose	↑ Nrf2-Keap1 activation, impaired autophagy; no GST/GGT antioxidant-cytotoxic response at low dose	[28]
	MDA-MB-453, MDA-MB-231, BT-474	phenolic-rich fractions from dark sweet cherry juice standardized for anthocyanins and procyanidins	MDA-MB-453: IC_50_ 83 µg/mL MDA-MB-231: IC_50_ 281 ± 56 µg/mL MDA-BT-474: IC_50_ 289 ± 19 μg/mL	[29]
	MCF-7	ethanolic and ethyl acetate extracts	IC_50_ 2.4 µg/mL(ethanolic), 2.9 µg/mL (ethyl acetate)	[17]
*P. cerasus*	A-549, THP-1, MCF-7, PC-3, NCI-H322	methanolic extract, a mixture of phenolic acids, flavonoids, and isoflavonoids	IC_50_ (NCI-H322) 5.59 µg/mL, IC_50_ (MTT): 5 µg/mL	[20]
*P. domestica*	C2C12	methanolic polyphenols fraction of the fruits	IC_50:_ 100 µg/mL	[21]
	Hep2c	plum wine-ethanolic extract of fruits standardized for total phenols 1.65 ± 0.07–2.18 ± 0.04 g/L	IC_50_ 27.29 ± 0.72 µg/mL	[30]
	RD	plum wine-ethanolic extract of fruits	IC_50_ 24.53 ± 0.94 µg/mL	[30]
	L2OB	plum wine-ethanolic extract of fruits	IC_50_ 32.28 ± 3.10 µg/mL	[30]
	AGS	neutral aqueous extract of plum fruit	IC_50_ 46.7–186 µg/mL	[31]
	SW-620	neutral aqueous extract of plum fruits	IC_50_ 46.7–186 µg/mL	[31]
	AGS	acidic extract of plum fruit	IC_50_ > 500 µg/mL	[31]
	SW-620	acidic extract of plum fruits	IC_50_ > 500 µg/mL	[31]
	MDA-MB-435	ethanolic extract of fruits standardized for flavonoids, procyanidins, phenolic acids, and anthocyanins	IC_50_ 54 ± 8 mg/L	[32]
	MCF-7	ethanolic extract of fruits	IC_50_ 925 ± 70 mg/L	[32]
	MCF-10A	ethanolic extract of fruits	IC_50_: 223 ± 103 mg/L	[32]
*P. mahaleb*	MCF-7	ethanolic fruit extract of (fruit juice and fruit peel, pulp and seeds)	no significant anti-proliferative or cytotoxic effect of the extract on MCF-7 in the concentration range from 0.9 mg/mL to 14.4 mg/mL, weak activity with concentration over 18.4 mg/mL	[33]
*P. persica*	MDA-MB-435	methanolic extract of fruits	IC_50_ 42 ± 4 mg/L	[32]
	MCF-7	methanolic extract of fruits	IC_50_ 515 mg/L	[32]
	MCF-10A	methanolic extract of fruits	IC_50_ 130 ± 36 mg/L	[32]
	MDA-MB-435	methanolic extract from fruits	decreased the tumor growth rate at the highest dose (0.8–1.6 mg/day), inhibited tumor growth and lung metastasis in vivo, through suppression of MMP-2, MMP-3, and MMP-13 gene expression	[23]
*P. serrulata*	HaCaT	cyanidin-3-O-glucoside and cyanidin-3-O-rutinoside from ethanolic extract of fruits	reduced the blue light-induced cytotoxicity	[7]
*P. serotina*	DU-145	methanolic extract of fruits standardized for total polyphenols: 367 ± 7.8 mg GAE/100 g dry fruits, total flavonoids: 59.4 ± 2.9 mg rutin/100 g dry fruits	~55% cell death was observed at 100 µg/mL	[34]
*P. spinosa*	HCT116	ethanolic extract of fruits	induced apoptosis in 44.8% of cells, with minimal necrosis (6.7%) compared to controls; apoptosis induced at rates comparable to 5-fluorouracil treatment	[24]
	colorectal cancer (3D model)	ethanolic extract of fruits	cell viability decreased in a 88.5% at 2 mg/mL, 77.2% at 4 mg/mL, and 73.2% at 6 mg/mL	[25]
	PC-3	ethanol extract from fruits standardized for β-sitosterol, campesterol and stigmasterol	IC_50_ 220 ± 0.63 µg/mL (PC-3)	[35]
	DU145	ethanol extract from fruits	IC_50_ 216.91 ± 28.16 µg/mL	[35]
	HT-29	aqueous extract from fruits (ultrasonic extraction for 30 min)	IC_50_ 123.8 µg/mL	[36]
	GBM	methanolic extract from fruits standardized for total phenolic content 2548 ± 18 mg GA/100 g	IC_50_ 50–63% viability	[37]
	AGS and KATO III	fruit cold-press extracted juice standardized for neochlorogenic acid, cyanidin-3-glucoside, rutin, gallic, caffeic, and vanillic acids	IC_50_ 65.63 for AGS and 69.76 μg/mL for KATO III cellsapoptotic/autophagic activity: ↑ caspase-3, Bax, LC3B-II; ↓ Bcl-xL, Beclin-1	[38]
	K562	aqueous and ethanol extracts from fruits	IC_50_ > 2000 µg/mL for both types of extracts	[39]
	HeLa	aqueous extract from fruits standardized for total phenol 23.19 ± 2.52 mg GAE/g of dry extract and total flavonoid 2.96 ± 0.22 mg QE/g of dry extract	IC_50_ 770 ± 4.24 µg/mL	[39]

Human cell lines: A-2058 melanoma; A-549 lung carcinoma; AGS gastric carcinoma; AMJ-13 Iraqi breast cancer;. BT-474 breast ductal carcinoma; Caco-2 colorectal adenocarcinoma; Colo-320 colon adenocarcinoma; Colo-741 colon cancer; DU145 prostate carcinoma; GBM glioblastoma multiforme; HaCaT keratinocytes; HeLa cervical cancer; HCT116 colorectal carcinoma; Hep-2 or Hep2c laryngeal carcinoma; Hep3B hepatocellular carcinoma; HepG2 hepatocellular carcinoma; HRT-18 colorectal adenocarcinoma; Huh-7 hepatocellular carcinoma; HT-29 colorectal adenocarcinoma; K562 chronic myelogenous leukemia; L2OB lung cancer; MCF-10A breast epithelial; MCF-7 breast adenocarcinoma; MDA-MB-231 breast cancer; MDA-MB-435 melanoma; MDA-MB-453 breast carcinoma; NB4 promyelocytic leukemia; NCI-H322 non-small cell lung carcinoma; OSCC (Scc-25) oral squamous cell carcinoma; PC-3 prostate cancer; RD rhabdomyosarcoma; SKOV-3 ovarian carcinoma; SR T-cell leukemia; SW-620 colon adenocarcinoma; T-24 bladder carcinoma; T47D breast ductal carcinoma; THP-1 monocytic leukemia; U937 histiocytic lymphoma. Murine cell lines: B-16 or B16F10 melanoma; C2C12 myoblast (muscle precursor); Hepa 1–6 liver cancer; RAW 264.7 macrophages; ↑ increase; ↓ decrease.

### 4.3. Antioxidant Activity

The antioxidant properties of *Prunus* species have been extensively investigated, particularly in *P. domestica*, and *P. armeniaca*. No data was found for *Prunus cerasoides*. The details of the reviewed experiments are presented in Supplementary Materials in Table S1, while some interesting results are discussed below.

Apricot samples demonstrated pronounced antioxidant capacity in all assays conducted, as indicated by DPPH IC_50_ values ranging from 21.32 ± 11.17 to 92.03 ± 12.02 µg/g FW. Sheikh et al. [20] found that the IC_50_ values for the apricot fruit extracts standardized for chlorogenic acid, rutin, quercetin, kaempferol, isorhamnetin, genistein, daidzin, formononetin, catechin, p-coumaric acid, and gallic acid, as characterized by LC-ESI-Q-TOF-MS analysis varied between 21.32 ± 11.17 to 92.03 ± 12.02 μg/g FW. The Irani variety exhibited the highest radical scavenging ability, with an IC_50_ value of 21.32 ± 11.17 μg/g FW, followed by the Tilton variety at 32.03 ± 20.56 μg/g FW. Wani et al. [40] conducted a study on methanol extracts from dried apricot fruit, optimized and standardized for total polyphenols 10.702 mg GAE/g, demonstrating an antioxidant potential of 91.16%. In a study from Turkey, ethanolic extracts of *Prunus armeniaca* showed considerable antioxidant potential, reaching 188.89 ± 2.02 mg TE/g in the DPPH assay and 267.03 ± 1.66 mg TE/g in the FRAP assay. The extracts were standardized for salicylic acid, kaempferol, fumaric acid, gallic acid, caffeic acid, and naringenin content [26].

*Prunus avium* showed moderate antioxidant activity, with DPPH inhibition ranging from ~30% to ~87% and an IC_50_ of 21.88 ± 0.32 µg/mL. Beconcini et al. [41] investigated the use of *P. avium* extract encapsulated in nanoparticles (NPs) derived from chitosan derivatives to enhance the bioavailability and protective effects of the extract on human umbilical vein endothelial cells (HUVECs) against oxidative stress. The cherry extract was prepared from frozen fully ripe *Prunus avium* fruits by homogenization with 70% acetone, followed by sonication, centrifugation, filtration, solvent removal, and finally freeze-drying to obtain a dry extract then standardized for total polyphenols (402.5 ± 8.4 mg GAE/100 g fresh weight (FW). The results demonstrated that nanoparticles based on a thiolated chitosan derivative not only improved the permeation of the extract, but also provided superior protection against oxidative stress. In another study, the antioxidant effect of six traditional and five commercial cultivars of sweet cherry was investigated. The findings indicated that the antioxidant activity for traditional cultivars ranged from 128.9 ± 3.38 to 632.3 ± 6.12, while the commercial cultivars exhibited higher antioxidant activity, ranging from 326.5 ± 79.1 to 692.9 ± 5.88 mmol Trolox per 100 g fresh weight. Importantly, a positive correlation between antioxidant activity measured by the DPPH assay and the concentration of fruit phenolics and β-carotene was noted [42].

*Prunus cerasus* fruits exhibited low antioxidant activity, with DPPH values ranging from 115 to 985 µmol TE/100 g FW. Ferretti et al. [43] observed a difference on the antioxidant activity of partially and fully ripened fruits of *Prunus cerasus* standardized for total phenol content 217 ± 50 mg GAE/100 g FW and 311 ± 44 mg GAE/100 g FW, respectively. The findings indicate that fully ripened fruits exhibit a higher ORAC value, compared to partially ripened fruits (2.54 ± 0.22 vs. 1.96 ± 0.25 mmol TE/100 g FW) and also a better score in DPPH radical scavenging activity (157 ± 18 vs. 115 ± 21 µmol TE/100 g FW). A sole in vivo study described the impact of sour cherries (4 mL of 10% and 50% dilution of original cherry juice vs. placebo) on the antioxidant potential in mice (n = 20). The juice was standardized for cyanidin-3-glucoside, cyanidin-3-rutinoside, pelargonidin-3-glucoside and pelargonidin-3-rutinoside. The findings indicated that sour cherry juice significantly elevated superoxide dismutase (SOD) activity in erythrocyte lysates (*p* < 0.002) compared to the control group. However, no significant changes were observed in catalase (CAT) and glutathione peroxidase (Gpx) activity in the erythrocyte lysates. In the liver, a notable increase in total SOD activity was recorded with the consumption of sour cherry juice (*p* < 0.001) relative to the control. CAT activity in the liver remained unaffected by sour cherry juice, while Gpx activity showed a significant increase in the liver of mice that were administered sour cherry juice (*p* < 0.003) compared to the control group [44].

*Prunus domestica* showed moderate DPPH antioxidant activity (IC_50_ = 34.28 ± 2.08 µg/mL), with skin and flesh IC_50_ values of 5.19 µg/mL and 5.95 µg/mL. Dhingra et al. [45] demonstrated that the total antioxidant capacity of *P. domestica* fruits were significantly influenced by its ethyl acetate fraction, which contains high levels of phenolic and flavonoid compounds that show maximum antioxidant activity, measured at 205.82 ± 2.52 to 1554.15 ± 5.73 µg AAE/mL. The extract was standardized for total phenolic content 33.88 ± 0.18 mg GAE/g and total flavonoid 47.96 ± 0.36 mg quercetin/g. Another study reported that among the neutral plum extracts standardized for 41 phenolics compounds, including: 11 flavan-3-ols, 14 flavonoids and 16 hydroxycinnamic acids and derivatives, Methley variety flesh exhibited the highest antioxidant capacity with an IC_50_ of 4.39 µg/mL, followed closely by the Satsuma cultivar skins with an IC_50_ of 4.85 µg/mL [31]. Similarly, Ozzengin et al. [46] demonstrated the antioxidant activity of *P. domestica* extract for two cultivars, Karaca and Üryani. The results indicated a FRAP value of 19.36 ± 8.66 mmol Fe(II) E/g for the Karaca cultivar and 23.74 ± 7.12 mmol Fe(II) E/g for Üryani. Additionally, the DPPH values were reported as 4.19 ± 1.11 mg TE/g for Karaca and 1.03 ± 0.32 mg TE/g for Üryani. The extracts were standardized for both cultivars, Karaca and Üryani and total phenolic content 833.6 ± 322.58 and 921.84 ± 239.00 mg/kg, respectively. Total anthocyanins content were 182.5 ± 110.92 and 106.22 ± 59.63 mg/kg for Karaca and Üryani cultivars.

Antioxidant activities of the extracts from different parts of *Prunus mahaleb* were evaluated by DPPH radical scavenging capacity assay. The extraction method used 3 × 300 mL of n-hexane at room temperature, then 3 × 300 mL methanol. The results indicated that hydrophilic extracts exhibited superior antioxidant activity compared to lipophilic extracts. Notably, the fruit extract demonstrated the highest antioxidant activity at 86.25%, comparable to the reference BHA solution, followed in order by the seed kernels, leaves, branches, and fruit stalks [47].

*Prunus persica* showed strong antioxidant activity (DPPH IC_50_: 0.155 mg/mL for butanol, 1.008 mg/mL for hexane fraction), with high DPPH (93.65–94.63%) and ABTS (148.21 mg TE/g) values. Giovanoudis et al. [48] compared the antioxidant activity of both ripe and unripe *Prunus persica* fruits of different cultivars. The unripe peach of the ‘Andross’ cultivar exhibited a total phenolic content (TPC) of 1465.32 mg of gallic acid equivalents per kilogram (mg GAE/kg). The antioxidant screening tests using FRAP and DPPH methods yielded results of 7.33 mmol and 5.12 mmol of ascorbic acid equivalents (AAE/kg), respectively. In contrast, the unripe fruit from the ‘Everts’ cultivar demonstrated an even higher antioxidant capacity, with a TPC of 1714.53 mg GAE/kg. Notably, the FRAP and DPPH values for this cultivar were recorded at 8.57 mmol AAE/kg and 6.08 mmol AAE/kg, respectively. The antioxidant activity of ethanolic extracts from the pulp, peel, and seed of *Prunus persica* was compared. Standardized for phenols content, the pulp exhibits 921.8 mg 151 chlorogenic acid equivalent/100 g FW, followed by the peel 448.6 FW and the seed 111.3 FW. The extract from the pulp exhibited the highest DPPH scavenging activity, showing an IC_50_ value of 12.0 µg/mL, followed by the peel extract with an IC_50_ value of 45.3 µg/mL. A similar pattern was observed with the ABTS radical, where the pulp and peel extracts demonstrated values of 6.8 and 8.4, respectively. The FRAP assay was employed to evaluate the reducing capacity of Tabacchiera peach, revealing that both pulp and peel extracts were the most active, with values of 30.2 and 78.9 µM Fe(II)/g, respectively. Additionally, the reducing ability of both extracts showed a significant correlation with phenolic levels (*p* < 0.01, r = 0.99) [49]. A study by Mihaylova et al. [50] investigated the DPPH activity of five different peach varieties. The extracts were standardized for total polyphenolic content, total flavoinod content, total monomeric anhocyanins, protocatechuic acid, chlorogenic acid, p-coumaric acid, sinapic acid and total phenolic acids. The results revealed significant variations among the extracts and varieties, ranging from 31.89 ± 0.31 to 728.98 ± 3.74 µMTE/100 g fw. The varieties “Filina” and “July Lady” demonstrated the highest DPPH inhibitory potential in the methanol extracts, while “Filina” and “Gergana” exhibited notable results for the water extract, with values of 180.01 ± 1.39 and 53.37 ± 0.30 µMTE/100 g fw, respectively. The results of a sole in vivo study involving rats (n = 24) demonstrated that fresh peach pulp and peel provide greater protection against cytotoxicity and oxidative stress compared to preserved peach pulp in most tissues. The extracts were checked for total phenolics content, all-trans-lutein, zeaxanthinin, β-cryptoxanthin, α-carotene, and β-carotene [51].

### 4.4. Anti-Inflammatory Activity

Among the *Prunus* species, *P. avium* fruits and by-products [52,53] have been extensively investigated. The majority of studies were performed in vitro, employing macrophage (RAW264.7) and microglial (BV2) cell lines to assess nitric oxide production, cytokine expression, and COX activity. Several in vivo and human studies were also conducted, particularly with *P. cerasus* [54], *P. persica* [47] and *P. spinosa* [55], confirming their anti-inflammatory effects through modulation of NF-κB, MAPK, and TLR4 pathways. No data on anti-inflammatory activity was so far published for fruits of *Prunus armeniaca* and *P. padus*. Various types of extracts—aqueous, hydroethanolic, and ethanolic—were used, all demonstrating consistent anti-inflammatory activity. The details of the reviewed experiments are presented in Table 2, while some interesting results are discussed below.

Anti-inflammatory properties of *P. avium* fruits were manifested as a reduction in C-reactive protein (CRP), regulated upon activation, normal T-cell expressed and secreted (RANTES), and nitric oxide (NO). The cherries were analyzed for hydroxycinnamates, anthocyanins, procyanidins, flavanols, total phenolics and dehydroascorbic acid [56]. Another study has examined the impact on COX-1 and COX-2. The activity of the fruits was significantly important, with IC_50_ 10.1 ± 0.8 to mg/mL and 16.9 ± 0.7 mg/mL, for COX-1 and COX-2, respectively. The extracts were standardized for phenolic acids, flavonols, flavanols, and anthocyanins [53].

A sole study reported anti-inflammatory properties of *P. cerasoides* fruit extract, manifested as a significant inhibition of nitric oxide (NO) production, reactive oxygen species (ROS), inflammatory cytokines (TNF-α, IL-6, IL-1β), inducible nitric oxide synthase (iNOS), and NF-kB in macrophages treated with the extract. Additionally, splenic lymphocytes treated with the extract displayed a reduction in Con-A-induced cell proliferation and a decrease in the number of CD3+CD4+ T cells [12].

Interestingly, although no data exists on *Prunus cerasus* anti-inflammatory in vitro properties, two studies described its effect in animals and humans. Šarić et al. [44] investigated the anti-inflammatory effects of sour cherry in a mouse model. The experimental and control groups, each consisting of 10 mice, were fed for 14 days with either commercial food pellets (control) or pellets mixed with 10% or 50% diluted *Prunus cerasus* cv. Maraska sour cherry juice (experimental). The results indicated that a 10% concentration of sour cherry juice led to a 33% reduction in COX-2 activity, while a 50% concentration resulted in a 41% decrease in COX-2 activity compared to the control group. Another study demonstrated a positive effect of *Prunus cerasus* fruit concentrate on physiological indicators in humans related to uric acid activity and inflammation. The extracts were analyzed for cyanidin (3.34 mg/mL), malvidin (4.69 mg/mL), pelargonidin (0.43 mg/mL), peonidin (0.22 mg/mL), delphinidin (0.169 mg/mL), petunidin (0.26 mg/mL). Twelve healthy participants were enrolled in a single-blind, randomized 48 h study, where they received 30 or 60 mL doses of CherryActive (Montmorency tart cherry supplement) mixed with water. The results indicated a reduction in uric acid activity and a decrease in high-sensitivity C-reactive protein (hsCRP) levels [54].

Silvan et al. [57] investigated the anti-inflammatory activity of *Prunus domestica* juice extracts analyzed for phenolic acids, flavonols, and anthocyanins and demonstrated an inhibitory capacity of 24–39% in the murine macrophage cell line RAW 264.7.

A sole study described that fruit extract from *P. mahaleb* exhibited notable anti-inflammatory properties in vitro. The findings revealed that the expression levels of ICAM-1, VCAM-1, and E-selectin were low in unstimulated human umbilical vein endothelial cells (HUVEC) but were significantly increased upon stimulation with lipopolysaccharide (LPS). Furthermore, the extract demonstrated a concentration-dependent reduction in the expression of ICAM-1, VCAM-1, and E-selectin, with significant effects noted even at a concentration of 1 mg/mL [33].

**Table 2:** Anti-inflammatory activity of the fruits of *Prunus* species.

Species	Study Type	Model/Subjects	Extract/Preparation	Effects	Reference
*P. avium*	in vitro	RAW264.7 macrophages, BV2 microglia	aqueous, hydroethanolic, ethanolic extracts	↓ NO, ↓ cytokines, ↓ COX activity; COX-1 IC_50_ = 3.6 ± 0.3 mg/mL (Sumhit cultivar); COX-2 IC_50_ = 9.2 ± 0.3 mg/mL (Techlovan cultivar)	[52,53]
*P. avium*	human	healthy adults (n = 18), 28 days	fruit consumption 280 g/d	↓ CRP, ↓ RANTES, ↓ NO	[56]
*P. cerasoides*	in vitro	macrophages; splenic lymphocytes	hydroethanolic fruit extract	↓ NO, ↓ ROS, ↓ TNF-α, IL-6, IL-1β, ↓ iNOS, ↓ NF-κB; ↓ CD3+CD4+ T cells	[12]
*P. cerasus*	in vivo	20 mice, 14-day feeding	10% & 50% sour cherry juice	↓ COX-2 activity (33% and 41%)	[44]
*P. cerasus*	human	12 healthy participants	Montmorency concentrate (30/60 mL) once taken	↓ uric acid; ↓ hsCRP	[54]
*P. domestica*	in vitro	RAW264.7 macrophages	plum juice extract	24–39% inhibition of inflammatory response	[57]
*P. mahaleb*	in vitro	HUVEC cells (LPS-stimulated)	fruit extract	↓ ICAM-1, VCAM-1, E-selectin (from 1 mg/mL)	[33]
*P. persica*	in vitro	BV2 microglia; primary astrocytes	methanol extract	↓ NF-κB, ↓ MAPK, ↓ COX-2, ↓ NO, ↓ p65 translocation	[58]
*P. persica*	in vitro	human mast cells	ethanol extract	↓ histamine release; ↓ TNF-α & cytokines; NF-κB dependent	[59]
*P. persica*	ex vivo	rat organs of brain cortex, kidney & liver	fresh pulp & peel	↓ TNF-α, ↓ IL-1β	[51]
*P. padus*	in vitro	LOX, hyaluronidase	lipophilic extract	LOX IC_50_ = 12.8 µg/U; hyaluronidase IC_50_ = 22.0 µg/U	[60]
*P. spinosa*	in vitro	human neutrophils, PBMCs	hydroalcoholic extract & phenolic fractions	↓ ROS, ↓ TNF-α, ↓ elastase; ↑ IL-10; slight ↓ IL-6 & IL-8	[61]
*P. serotina subsp. capuli*	in vitro	RAW264.7 macrophages	fruit extract	↓ nitrite, ↓ IL-1β mRNA, ↓ TNF-α mRNA & secretion	[62]
*P. serrulata*	in vitro	RAW264.7 macrophages	Ag/Au nanoparticles from fruit extract	↓ NO, ↓ PGE2, ↓ iNOS, ↓ COX-2; ↓ LPS activation	[63]
*P. spinosa*	in vitro	various inflammatory cell models	ethanol extract	↓ ROS (Nrf2 activation); ↓ TLR4/NF-κB cascade	[64]
*P. spinosa*	in vitro	cell model (40 µg/mL)	ethanol extract	TLR4-mediated; modulation of miR-146a, IRAK-1, IL-6	[65]
*P. spinosa*	in vivo	*C. elegans*	fruit extract	↑ miR-146a; ↓ TLR-NF-κB; ↓ IL-6	[66]
*P. spinosa*	in vivo	60 rats (gastric ulcer model)	ethanol extract	↓ TNF-α, IL-6, IL-1β, IL-8, NF-κB (comparable to lansoprazole)	[55]

See Section Abbreviations for the explanation of the symbols used within the table; ↑ increase; ↓ decrease.

Methanol extracts from fruits of *Prunus persica* significantly reduced pro-inflammatory mediators and cytokines at the transcriptional level in LPS-stimulated microglial cells (BV2 cells), observed as the suppression of NF-κB and MAPK activation, as well as a decrease in cyclooxygenase-2 levels. The extract was analyzed for content of quercetin, quercetin-3-O-glucoside, catechin and chlorogenic acid. Additionally, the extracts decreased nitric oxide production and the translocation of p65 also in primary astrocytes [58]. Shin et al. [59] investigated the effects of ethanol extract (extracted twice with 70% ethanol at 70 °C for 3 h using a water bath) from the fruits of *Prunus persica* on mast cell-mediated allergic inflammation. The extract significantly inhibited both compound 48/80-induced systemic anaphylaxis and immunoglobulin E-mediated local allergic reactions in a dose-dependent manner. Furthermore, the extract was found to reduce histamine release from mast cells, which appears to be mediated through the modulation of intracellular calcium levels. Additionally, the extract diminished the expression and secretion of pro-inflammatory cytokines in human mast cells stimulated by phorbol 12-myristate 13-acetate and calcium ionophore A23187 (PMACI).

The inhibitory effect of this extract on pro-inflammatory cytokines was determined to be dependent on nuclear factor (NF)-κB signaling. Lipophilic-chloroform extracts from fruits of *Prunus padus* standardized for corosolic, ursolic and oleanolic acids, were more effective inhibitors of lipoxygenase (LOX) with IC_50_ values 12.8 µg/U, compared to hyaluronidase (IC_50_ 22.0 µg/U) [60]. Gasparotto et al. [51] conducted an ex vivo study which found that the release of tumor necrosis factor-α and interleukin-1β was significantly reduced with the intake of fresh peach pulp and peel, as opposed to preserved peach pulp.

A sole study described the effect of the fruits of the Capuli cherry’s (*Prunus serotina* Ehr. subsp. *capuli* (Cav.) McVaugh) oxidative damage and pro-inflammatory cytokine production in LPS-stimulated RAW 264.7 macrophages. The plant material was analyzed for anthocyanins, vitamin C and *β*-carotene. The findings indicated a significant decrease in oxidative damage markers, including nitrite levels, interleukin 1β messenger RNA levels, and tumor necrosis factor α messenger RNA levels and secretion, after pre-incubation with the Capuli extract and subsequent stimulation with LPS [62].

Singh et al. [63] investigated the anti-inflammatory effects of silver and gold nanoparticles derived from *P. serrulata* fresh fruit extract. The extract was analyzed for total phenolic contents and total flavonoids content. The findings indicated a decreased expression of inflammatory mediators, including nitric oxide (NO), prostaglandin E2 (PGE2), inducible nitric oxide synthase (iNOS), and cyclooxygenase-2 (COX-2) in lipopolysaccharide (LPS)-induced RAW 264.7 cells. Additionally, the nanoparticles exhibited a significant suppression of LPS-induced activation.

Colomba et al. [64] demonstrated that the ethanol extract of the fruits of *P. spinosa* exerted a cytoprotective effect through its antioxidant properties. This was achieved by reducing reactive oxygen species (ROS) levels during inflammation, particularly when the Nrf2 pathway was activated. Furthermore, the extract prevented a pro-inflammatory response by inhibiting the TLR4/NF-kB-mediated inflammatory cascade. The extracts were analyzed for hydroxycinnamic acid derivatives, hydroxybenzoic acid derivatives, flavonoid derivatives and anthocyanins. Hydroalcoholic extract and phenolic-enriched fractions of *P. spinosa* fruits demonstrated a significant capability to modulate the pro-oxidant, pro-inflammatory, and anti-inflammatory responses in human neutrophils and peripheral blood mononuclear cells (PBMCs). The extract was analyzed for gallic acid, phenolic acids (caffeoyl-, coumaroyl-, and feruloylquinic acids), flavonoids (mostly quercetin mono-, di- and triglycosides), condensed proanthocyanidins, and anthocyanins (cyanidin and peonidin glycosides). Specifically, a strong downregulation in the release of reactive oxygen species, tumor necrosis factor-alpha (TNF-α), and neutrophil elastase, alongside an upregulation in the secretion of interleukin-10 (IL-10) was observed. Additionally, there was a slight inhibition in the production of interleukin-8 (IL-8) and interleukin-6 (IL-6) in the cells stimulated with fMLP, fMLP + cytochalasin B, and lipopolysaccharide (LPS) [61]. Sabatini et al. [65] demonstrated that an ethanol extract of *Prunus spinosa* fruit at a concentration of 40 µg/mL exhibited in vitro significant anti-inflammatory activity mediated through TLR4 signaling, involving the expressions of miR-146a, IRAK-1, and IL-6. It was observed that during a pro-inflammatory challenge, the downregulation of miR-146a results in an increased expression of its target proteins, IRAK-1 and IL-6. The extract was analyzed for hydroxybenzoic acids, flavanols, anthocyanins: cyanidin 3-rutinoside and cyanidin 3-glucoside, and hydroxycinnamic acids.

Coppari et al. [66] investigated the effects of *P. spinosa* fruit extract on worms. The extract was analyzed for phenolic acids, anthocyanins, flavonoids (flavonols and their glucosides). The extract upregulated intracellular miR-146a, observed as a downregulation of the TLR-NF-κB-mediated inflammatory response, primarily by inhibiting the TLR4 signaling pathway and reducing cytokine production, specifically interleukin-6 (IL-6). Additionally, a decrease in the expressions of IRAK-1 and IL-6 was noted, providing strong evidence for the extract’s anti-inflammatory activity. In another study, *Prunus spinosa* ethanol extract in a rat model (n = 60) of indomethacin-induced gastric ulceration significantly reduced the levels of TNF-α, IL-6, IL-1β, IL-8, and NF-kB, with the effect comparable to the reference substance lansoprazole. The extract was analyzed for phenolic acids, flavonoids, anthocyanins and coumarin [55].

### 4.5. Gastrointestinal Disorders

Some of the reviewed studies focused on the activities directed to gastrointestinal disorders. These included the problems of diabetes, metabolic disorders, but also the hepatoprotective effect. The results of the studies, performed in vitro, in vivo and human, should be of special interests, taking into account that *Prunus* fruits are edible, and in most cases are included in daily diet, what may translate into their support in the prevention of some diseases.

#### 4.5.1. Impact on Metabolic Disorders

*Prunus avium* fruits’ extract standardized for hydroxycinnamic acids and cyanidin-3-O-rutinoside inhibited α-glucosidase in vitro, however the effect was few times weaker than the reference acarbose (IC_50_ 53.15 ± 1.32 vs. 389.89 ± 4.01 µg/mL) [27]. Another study indicated the in vitro impact of *P. avium* fruits on the inhibition of α-amylase and α-glucosidase with the IC_50_ values 46.7 ± 0.4 to 78.3 ± 1.4 mg/mL, and 4.4 ± 0.2 to 10.5 ± 1.0 mg/mL, respectively. The authors used Pearson’s correlation and Principal Components Analysis (PCA) to show that phenolic compounds, minerals, and amino acids were the main contributors to in vitro enzymatic activity [53]. A single-blind randomized placebo-controlled study verified the effects of *P. avium* juice in obese adults. The juice was analyzed for total phenolics and anthocyanins content. Participants consumed either 200 mL of a dark sweet cherry beverage (n = 19) or a placebo beverage (n = 21) twice daily over a 30-day period. The findings indicated no significant differences between the groups concerning blood lipids, glucose levels, or liver enzyme activity, but taking the juice was associated with a reduction in blood pressure levels and inflammation [67]. Similarly, Kelley et al. [56] conducted a study on healthy participants (n = 18) and supplemented their diets with 280 g of Bing sweet cherries daily for a duration of 28 days (no placebo group). The results indicated no significant improvement in cholesterol parameters.

Although no data exists on the in vitro antidiabetic effect of *P. cerasus*, a sole study investigated the effects of sour cherry juice (standardized for total anthocyanins content) on blood glucose levels and cardiovascular risk factors in diabetic women (n = 19). After six weeks of consuming 40 g of the juice, significant reductions were observed in body weight (*p* < 0.01), blood pressure, and HbA1c levels (*p* < 0.05). Additionally, total cholesterol and LDL-C levels decreased significantly in a subgroup of patients with LDL-C levels exceeding 100 mg/dL [68]. Two other studies were performed on the impact of *P. cerasus* on metabolic disorders. One of them examined the effects of tart cherry seed powder and juice on metabolic syndrome in rats (n = 44). The plant material was standardized for anthocyanins. The seed powder was administered at a dosage of 0.1 mg per gram per day, along with juice containing 1 mg of anthocyanins, and two control groups with and without dietary restriction for 5 weeks. The results indicated that both the seed and juice groups experienced reductions in blood pressure and glycemia when compared to the placebo group. Additionally, the consumption of tart cherry was found to modify aquaporin-4 and endothelial inflammatory markers [69]. Kimble et al. [70] conducted a randomized, placebo-controlled parallel study examining the impact of tart cherry concentrate supplementation over a three-month period on cardiometabolic risk factors in middle-aged adults (n = 50). The Tart Montmorency cherries concentrate was analyzed for total phenolics and anthocyanins. Participants consumed 30 mL of their assigned treatment twice daily over a three-month period, receiving either cherry juice or an isocaloric placebo. The findings indicated no discernible effect of the intervention on vascular function or metabolic health variables among the participants following the study period.

Rybak et al. [71] demonstrated the inhibitory effects in vitro on α-amylase activity of forty-three cultivars of *Prunus domestica*, with IC_50_ values ranging from 2.63 to 61.53 mg/mL, weaker than the reference inhibitor acarbose (IC_50_ 0.01–0.2 mg/mL). In the case of α-glucosidase, the cultivars showed IC_50_ values ranging from 0.19 to 24.07 mg/mL, whereas acarbose exhibited inhibitory activity within the range of 0.01 to 0.5 mg/mL. Pancreatic lipase inhibition was observed with IC_50_ values from 0.50 to 8.20 mg/mL, compared to the reference inhibitor orlistat, with IC_50_ values between 0.02 and 0.06 mg/mL. Additionally, inhibition of lipoxygenase (15-LOX) was reported with IC_50_ values ranging from 4.19 to 32.67 mg/mL, while standard inhibitors such as nordihydroguaiaretic acid or zileuton demonstrated IC_50_ values in the range of 0.1 to 1.0 µg/mL. The extract was analyzed for phenolic acid, flavonols, procyanidins and anthocyanins. Additionally, a human study investigated the effects of dried plums (*P. domestica*) in patients with hypercholesterolemia. The plant material was standardized for total polyphenol contents. Forty-eight participants were provided with 100 g of prunes daily for a duration of six weeks. The results indicated a significant reduction in total cholesterol levels, as well as a decrease in the LDL fraction and the LDL/HDL atherogenicity index [72].

A sole study evaluated in vitro antihyperglycemic effect of the water extract of *P. mahaleb* fruits analyzed for content of total phenols, flavonoids, and tannins. The results indicated an IC_50_ value of 3.44 ± 0.14 mg/mL for α-amylase and 1.35 ± 0.04 mg/mL for α-glucosidase inhibition, but no reference drug was used in the study [73].

Marčetić et al. demonstrated enzyme in vitro inhibitory effects of *P. spinosa* fruits’ methanol extracts on α-amylase and α-glucosidase, with IC_50_ values ranging from 1.26 ± 0.04 to 2.05 ± 0.05 mg/mL and 0.43 ± 0.06 to 0.63 ± 0.02 mg/mL, respectively. The extracts were analyzed for total phenolic, total flavonoid, total anthocyanin [74]. Another study reported the inhibitory activity of ethanol extracts from the fruits, demonstrating a potent effect on α-glucosidase inhibition, with an IC_50_ value of 199.84 µg/mL, which was found to be higher than that of the standard drug acarbose. The ethanolic extract was analyzed for total phenolic content, total flavonoid content, flavonol, phenolic acid, monomeric anthocyanins and total anthocyanins content [75].

#### 4.5.2. Hepatoprotective Activity

A sole animal study demonstrated that a diet containing 10–20% apricot provided a protective effect against carbon tetrachloride (CCl_4_)-induced hepatic steatosis and liver damage in rats (n = 42). All groups receiving apricots were fed with a diet containing 10% or 20% apricots for a period of five months. The CCl_4_ administration was performed on the relevant groups at a dosage of 1 mg/kg over three days at the conclusion of the five-month period. The inclusion of apricot in the diet reduced oxidative stress, observed as the significant improvement in malondialdehyde and total glutathione levels, as well as in the activities of catalase, superoxide dismutase, and glutathione peroxidase, and also improved histological damage [76].

A study on the effects of a dry extract from *Prunus domestica* fruits in rats (n = 40) with constipation and alcohol-induced liver damage was performed. The examined extract was administered orally at a dose of 200 mg/kg, and the reference hepatoprotective agent Silybor at a dose of 25 mg/kg, with or without induced constipation, over a period of 10 days. The results indicated significant improvements in liver damage markers and demonstrated laxative activity [77]. A similar study on the hepatoprotective effects of *P. domestica* fruit extract in rats (n = 24) was performed. The extract of Prunofit was analyzed for carboxylic acids: citric, malic, oxalic, and succinic acids. The reference and experimental groups of animals with alcoholic hepatitis were administered the standard treatment, methionine, at a dose of 155 mg/kg, and plum extract at a dose of 200 mg/kg, respectively. The findings indicated a reduction in the intensity of lipolysis processes, fatty hepatosis, and manifestations of hyperlipidemia. Additionally, the study showed a decrease in the levels of total lipids, cholesterol, triacylglycerols, and free fatty acids [78].

Likewise, the hepatoprotective effect of a flavonoid-rich concentrated extract from fruit of *Prunus mahaleb* was examined in mice (n = 24). The extract was not analyzed for phytochemical composition. The experimental group received daily oral supplementation with the fruit concentrate for four weeks. The administered dose was 25 µL/kg bw, which is equivalent to approximately 6.2 g of fresh fruit per day when converted to a human equivalent dose. The control group did not receive this supplementation. The findings indicated that the administration of the extract led to an enhancement in the liver’s antioxidant defenses, which was correlated with increased oxidative metabolism and elevated levels of peroxisome proliferator-activated receptor gamma coactivator 1-alpha (PGC-1alpha) and heme oxygenase-1 (HO-1), when compared to the negative control group [79].

The effects of fruit of *P. serotina* powder and extract on liver injury induced by acetaminophen in rats (n = 30) was also examined. The control group and three treatment groups of rats were administered the following substances: silymarin at a dosage of 100 mg/kg/day, 10% dried black cherry, and black cherry extract at a dosage of 500 mg/kg/day, for 28 days. The findings from the intervention indicated a reduction in liver enzymes and significant decreases in liver malondialdehyde (MDA) levels. Furthermore, the study observed notable increases in hepatic catalase (CAT) activity and glutathione (GSH) content [80].

### 4.6. Urinary Tract Disorders

*Prunus cerasoides* and *P. cerasus* fruits were examined towards their antiurolithiatic potential. Bawari et al. investigated the hydroethanolic extract of *Prunus cerasoides* fruits in relation to calcium oxalate urolithiasis. The extract was analyzed for total phenolic and flavonoid content. The in vitro screening was conducted using nucleation and aggregation assays, while preclinical evaluations were performed on male Wistar rats (n = 42) with urolithiasis induced by ethylene glycol (0.75% *v*/*v*) and ammonium chloride (1% *w*/*v*). The extract of *P. cerasoides* fruit facilitated the nucleation of multiple small-sized calcium oxalate crystals and significantly inhibited their aggregation in metastable solutions. Furthermore, the extract effectively mitigated lithogenic treatment-induced anorexia, weight loss, polydipsia, hypocitraturia, and hypomagnesuria. It also demonstrated a preventive effect on the deposition of calcium, oxalate, and phosphate in kidney tissues, along with an ameliorative effect on serum levels of urea, creatinine, and uric acid. Additionally, the extract inhibited the oxidative stress-induced degeneration of renal tissue, as evidenced by histopathological analysis of the kidneys [81].

The effects of tart cherry (*P. cerasus*) juice on serum uric acid levels, hepatic xanthine oxidoreductase activity, and oxidative stress in both normal and hyperuricemic rat models were examined. The results indicated that tart cherry juice treatment effectively inhibited hepatic xanthine oxidase/dehydrogenase activity. Additionally, a significant increase (*p* < 0.05) in serum total antioxidant capacity was noted in the rats (n = 36) treated with tart cherry juice, across both the normal and hyperuricemic groups. Furthermore, the oral administration of tart cherry juice resulted in a significant reduction (*p* < 0.05) in malondialdehyde (MDA) concentration among the hyperuricemic rats. However, orally administered tart cherry juice did not result in a significant reduction in serum uric acid levels in normal rats after 14 days. In contrast, allopurinol, a known xanthine oxidase inhibitor, significantly decreased (*p* < 0.01) serum uric acid levels in the same population after the same period [82].

A single, open labeled and single armed human study to evaluate the effects of ethanolic extract of *Prunus domestica* (ProsmanTM) on benign prostate hyperplasia (BPH) was performed. In the study 140 male participants received 100 mg of (ProsmanTM) twice daily for a duration of 12 weeks. The results indicated a significant efficacy in BPH patients, as evidenced by a reduction in the International Prostate Symptom Score (IPSS), prostate volume, and serum prostate-specific antigen (PSA) levels [83].

### 4.7. Central Nervous System Disorders

Antognoni et al. [84] demonstrated that methanolic extract of the fruits of *P. avium* exhibited neuroprotective in vitro effect in neuron-like SH-SY5Y cells by mitigating oxidative stress and enhancing the expression of brain-derived neurotrophic factor (BDNF), associated with cell survival. The extract was analyzed for cyanidin-3-O-rutinoside, cyanidin-3-O-glucoside, peonidin-3-O-rutinoside, and phenolic acids [84]. Similar results were described in another study on methanolic sweet cherries extract, which revealed significant neuroprotective in vitro properties to neuronal cells (PC 12) with the induced oxidative stress-induced damage. The observed effect was dose-dependent, and primarily attributed to the presence of anthocyanins [85].

Neuroprotective in vitro effects were also noted for the water extract of *P. mahaleb* fruits, on hypothalamic HypoE22 cells. The cells were subjected to a pro-oxidant stimulus involving hydrogen peroxide at a concentration of 300 µM, while the extract effectively prevented the turnover of dopamine, as indicated by the DOPAC/DA ratio. This ratio serves as a valuable indicator of monoamine oxidase-B (MAO-B) activity, highlighting the neuroprotective effects of the fruits, which also contribute to the anti-neuroinflammatory properties elicited by the extract. Importantly, the extract was non-toxic to the cells within the effective concentration range [73].

Interesting in vivo studies on the inhibitory effects of water, methanol, and chloroform extracts of *Prunus persica* fruits (extracted with boiling water for 3 h) on acetylcholinesterase (AChE) activity in the brain and plasma of rats were performed. The findings indicated that the chloroform extract exhibited the most significant inhibitory effect, with IC_50_ value of 5.6 µg/mL. Furthermore, the oral administration of *Prunus persica* extract or tacrine resulted in a dose-dependent inhibition of cholinesterase activities in both the brain and plasma. The ID_50_ values for brain cholinesterase activity were found to be 2.7 g/kg for the extract and 8.9 mg/kg for tacrine. In contrast, the ID_50_ values for plasma cholinesterase activity were recorded at 18.6 g/kg for the extract and 27.5 mg/kg for tacrine. Consequently, the ratios of the ID_50_ values indicated that plasma cholinesterase activity was lower than that of brain cholinesterase, with ratios of 6.0 and 3.1, respectively. Unfortunately, no information on the number of animals used in the study was provided [86].

Gómez-Mejía et al. [87] conducted a study on hydroethanolic extract of sloe skin and seeds of *P. spinosa* to evaluate its neuroprotective potential in vitro against Alzheimer’s disease. The extract was analyzed for total phenolic content, with predominant content of quercetin, 2,3-dihydroxybenzoic and ferulic acids. This included the inhibition of beta-amyloid (Aβ42) aggregation in vitro and evaluated the cytoprotective effects against oxidative stress in the SH-SY5Y neuronal cell line. A multivariate analysis was employed to correlate the phenolic composition with the observed bioactivities, revealing a significant potential for further investigation.

Two human studies described the potential of *P. cerasus* in sleeping disorders. The effect of tart cherry juice on melatonin levels and overall sleep quality was examined, in a randomized, double-blind, placebo-controlled, crossover design. The volunteers (n = 20) consumed either a placebo or tart cherry juice concentrate for seven days. The results indicated a significant increase in total melatonin content in the cherry juice group, with no notable differences observed between the baseline and placebo trials. Additionally, there were significant improvements in time spent in bed, total sleep time, and sleep efficiency among participants who received the cherry juice supplementation [88]. Similar observations were noted by Pigeon et al. [89] in a randomized, double-blind, crossover design study on the effects of tart cherry juice (two 8-ounce servings for 14 days) on the sleep patterns of 15 older adults experiencing chronic insomnia. The findings indicated statistically significant improvements in all sleep variables from pre-treatment to post-treatment. However, these effects were notably less pronounced compared to established evidence-based treatments for insomnia.

## 5. Limitations of the Studies and Future Perspectives

It should be emphasized that the majority of the reviewed studies were performed in vitro, while only several described the effect in animal models (Table 3) and even fewer in humans (Table 4). Although some of the results from the in vitro studies seem to be interesting and provide perspective, much more effort should be made to verify the potential health benefits of different *Prunus* species in animal or human organisms. Moreover, some of the studies were focused on bioactivities that were not fully justified. This includes first of all the verification of their potential directed to some neurological disorders, the study of which is not well supported by the phytochemical composition of *Prunus* fruits.

Despite the proper phytochemical characterization of some of the extracts studied, only a few reports tried to link the observed pharmacological or biological effect with the amount of the phytochemicals detected or particular bioactive compound, which is a significant gap needed to be filled. Moreover, in some studies, the extracts examined were not standardized or even quantified to any of the phytochemicals present, which makes their results questionable, and not credible enough to draw further conclusions. As most of the extracts were rich in polyphenolic compounds (i.e., anthocyanins, phenolic acids, flavonoids) it can be assumed that at least some of the observed effects are connected to the presence of the phytochemicals. A more integrative interpretation of signaling pathways reported for the extracts of *Prunus* fruits may suggest that their biological activities result from the complementary and interconnected inflammatory, cell survival, and stress-response networks, rather than isolated molecular targets. A number of studies cited in the review often indicate TLR4/NF-κB, MAPK, and PI3K/Akt/mTOR signaling pathways as predominates. These pathways are functionally interconnected, with TLR4 acting as an upstream regulator of inflammation, NF-κB and MAPK coordinating transcriptional and stress responses, and Akt/mTOR referring to cell survival and metabolic adaptation. Evidence from the studies on different *Prunus* fruits highlights that polyphenol-rich extracts exert multitarget effects, simultaneously suppressing NF-κB-driven inflammation, modulating MAPK-dependent apoptosis, and inhibiting Akt/mTOR-mediated proliferation [18]. This multifaceted regulation underlies the broad spectrum of biological activities observed in *Prunus*, including anti-inflammatory, anticancer, and anti-metastatic effects.

Another limitation concerns the bioavailability of polyphenols present in *Prunus* fruits. Anthocyanins and polyphenols from *Prunus* fruits exhibit relatively low systemic bioavailability due to chemical instability, limited intestinal absorption, and extensive phase II metabolism. Following ingestion, these compounds are rapidly transformed into conjugated derivatives and microbiota-generated phenolic metabolites, which represent the predominant bioactive forms in circulation. Despite low plasma concentrations of parent compounds, accumulating evidence indicates that both conjugated and microbial metabolites contribute significantly to the observed biological effect [90]. Additionally, the relatively low systemic exposure may be compensated for by the repeated dietary intake of *Prunus* fruits, and their high anthocyanins content.

The majority of the in vitro studies did not include non-cancerous cells as the reference for the safety of the examined extracts. Likewise, reference compound was used only rarely in the reviewed studies, the use of which can enable us to evaluate the strength of the effect. Scarce animal and human studies reviewed do not allow us to draw valid final conclusions. Human trials are based on small study groups, often consisting of several individuals, and only one study was performed on a study group exceeding 100 participants. Moreover, in our opinion, most of the performed trials are of rather poor quality, with no placebo group included. Another aspect that can raise concerns is the quite short duration of the human trials, which may limit the observation of potential adverse effects. Similar critical concerns are present in the animal studies, where the relatively small number of animals in the groups and the lack of a control group make the results not credible.

Future investigations should prioritize the use of standardized and chemically characterized extracts, including the quantification of major phytochemicals and the application of appropriate quality-control procedures. In vitro studies should routinely include non-cancerous or normal cell lines to assess the selectivity and safety of tested extracts. Additionally, the use of reference compounds or established positive controls should become a standard practice, allowing direct comparison of efficacy and facilitating the evaluation of the biological relevance of observed effects. Among the reviewed species, fruits of *Prunus avium* and *P. domestica* were the most examined, while only scarce data exists for the fruits of *P. cerasoides* and *P. padus*. Moreover, the evidence from human studies indicates *P. cerasus* and *P. avium* fruits as quite potent in some metabolic disorders, although this issue needs further investigation.

There is also a need for more robust in vivo studies employing adequately sized animal groups, proper control groups, and well-designed experimental protocols. Such studies would provide more reliable evidence regarding efficacy, safety, pharmacokinetics, and mechanisms of action.

Most importantly, high-quality clinical trials are required to confirm the translational potential of promising phytochemicals and plant extracts. Future human studies should include larger and more diverse populations, randomized placebo-controlled designs, and longer follow-up periods to evaluate both efficacy and potential adverse effects. The implementation of rigorous methodological standards will enhance the reliability of clinical evidence and facilitate the development of evidence-based applications of these natural products.

## 6. Conclusions

Despite the increasing number of studies on the bioactivity of *Prunus* fruits, the so far obtained results cannot clearly state their health benefits that may have translational consequences. However, apart from the above-mentioned limitations and weaknesses, we believe that there is a future for the fruits, in terms of their use in chemoprevention or slowing down the risk of the development of some diseases. However, future studies are urgently needed, which should be focused on: (i) indicating the relationships between the phytochemical composition of *Prunus* fruits and their bioactivity; (ii) pharmacokinetic evaluation of the fruit extracts, particularly their absorption and metabolism in the gastrointestinal tract; (iii) in-depth mechanistic studies, related to the so far noted bioactivities; and (iv) the impact of the fruits on different disorders of the gastrointestinal tract, especially in terms of chemoprevention.

## Figures and Tables

**Table 3:** Animal studies performed with the fruits of *Prunus* species.

Activity	Species	Model (n)	Study Design	Effects	Reference
anticancer	*Prunus* *cerasus*	Swiss albino mice (38)	methanolic fruit extract; 200 mg/kg intraperitoneally; tumor models: Ehrlich Ascites Carcinoma (EAC) and methylcholanthrene-induced ascites (Meth-A) for 12 days	Tumor inhibition: 72.31% (EAC); 68.69% (Meth-A)	[20]
anticancer	*Prunus spinosa*	mice (30)	extracts at high dose (0.15 mg/mL), low dose (0.05 mg/mL), and control; oral gavage; once daily, 5 days/week, 1 month; colorectal cancer model; Trigno M nutraceutical activator	Significant delay in colorectal tumor growth vs. control	[24]
antioxidant	*Prunus* *cerasus*	mice (20)	4 mL of 10% or 50% diluted sour cherry juice vs. placebo for 14 days; assessment of erythrocyte and liver antioxidant enzymes	↑ SOD (erythrocytes *p* < 0.002; liver *p* < 0.001); ↑ liver GPx (*p* < 0.003); no CAT change	[44]
antioxidant	*Prunus persica*	rats (24)	fresh pulp and peel vs. preserved pulp; oxidative stress markers assessed in multiple tissues	Fresh fruit provided stronger cytoprotection and antioxidant effects	[51]
anti-inflammatory	*Prunus* *cerasus* (cv. Maraska)	mice (20)	14-day feeding with pellets mixed with 10% or 50% diluted juice; control group received standard pellets	↓ COX-2 activity by 33% (10%) and 41% (50%)	[68]
anti-inflammatory	*Prunus spinosa*	worm model	fruit extract; evaluation of miR-146a expression and TLR-NF-κB pathway signaling	↓ TLR4, IL-6, IRAK-1; NF-κB suppression	[66]
anti-inflammatory	*Prunus spinosa*	rats (60)	indomethacin-induced gastric ulcer model; ethanol extract; comparison with lansoprazole once taken	↓ TNF-α, IL-6, IL-1β, IL-8, NF-κB (comparable to reference drug)	[55]
anti-metabolic	*Prunus* *cerasus*	rats (44)	tart cherry seed powder (0.1 mg/g/day) + juice (1 mg anthocyanins); 5 weeks; control groups ± dietary restriction	↓ BP; ↓ glycemia; aquaporin-4 & endothelial inflammation modulation	[69]
hepatoprotective	*Prunus* *armeniaca*	rats (42)	10–20% apricot diet for 5 months; CCl_4_ (1 mg/kg) administered over 3 days at end	↓ MDA; ↑ GSH, SOD, CAT, GPx; improved histology	[76]
hepatoprotective	*Prunus* *domestica*	rats (40)	alcohol-induced liver damage ± constipation; 200 mg/kg extract orally; 10 days; Silybor (25 mg/kg) comparator	Improved liver enzymes; laxative activity	[77]
hepatoprotective	*Prunus* *domestica*	rats (24)	alcoholic hepatitis model; 200 mg/kg extract vs. methionine (155 mg/kg) for 72 h	↓ lipolysis, fatty hepatosis, lipids & TAG	[78]
hepatoprotective	*Prunus mahaleb*	mice (24)	25 µL/kg flavonoid-rich concentrate orally for 4 weeks	↑ antioxidant defense; ↑ PGC-1α & HO-1 expression	[79]
hepatoprotective	*Prunus* *serotina*	rats (30)	acetaminophen-induced liver injury; 10% dried fruit or 500 mg/kg extract; 28 days; silymarin comparator	↓ liver enzymes; ↓ MDA; ↑ CAT & GSH	[80]
urolithiasis	*Prunus* *cerasoides*	rats (42)	ethylene glycol (0.75%) + ammonium chloride (1%) urolithiasis model; hydroethanolic extract	↓ CaOx agregation; ↓ renal oxidative damage; improved renal markers	[81]
hyperuricemia	*Prunus* *cerasus*	rats (36)	hyperuricemia model; tart cherry juice; compared with allopurinol, no phytochemical composition analyzed	↓ XO activity; ↑ TAC; ↓ MDA	[82]
neuroprotective	*Prunus persica*	rats (n.g.)	water, methanol, chloroform extracts; oral administration; compared with tacrine; AChE inhibition assessed in brain & plasma	Dose-dependent cholinesterase ↓; chloroform extract IC_50_ 5.6 µg/mL	[86]

See Section Abbreviations for the explanation of the symbols used within the table; ↑ increase; ↓ decrease.

**Table 4:** Human studies performed with the fruits of *Prunus* species.

Activity	Species	Model (n)	Study Design	Effects	Reference
anti-inflammatory	*Prunus* *cerasus* (Montmorency)	humans (12)	single-blind, randomized 48 h study; no placebo, 30 or 60 mL CherryActive + 100 mL water once taken	↓ uric acid activity; ↓ hsCRP	[54]
antidiabetic	*Prunus* *cerasus*	diabetic women (19)	40 g sour cherry juice daily for 6 weeks; no placebo; metabolic & cardiovascular parameters assessed	↓ body weight (*p* < 0.01); ↓ BP; ↓ HbA1c (*p* < 0.05); ↓ LDL-C (subgroup)	[68]
anti-metabolic	*Prunus avium*	healthy adults (18)	280 g Bing cherries daily for 28 days; no placebo	no significant lipid improvement	[56]
anti-metabolic	*Prunus avium*	obese adults (40)	single-blind RCT; 200 mL dark sweet cherry beverage (n = 19) or placebo (n = 21) twice daily for 30 days	no lipid/glucose effect; ↓ BP & inflammatory markers	[67]
anti-metabolic	*Prunus* *cerasus*	adults (56)	randomized placebo (n = 28) controlled parallel trial; 30 mL concentrate twice daily (n = 28); 3 months	no effect on vascular function or metabolic markers	[70]
anti-metabolic	*Prunus* *domestica*	hypercholesterolemic adults (48)	100 g prunes daily; 6 weeks; no placebo, lipid profile assessment	↓ total cholesterol; ↓ LDL; ↓ LDL/HDL index	[72]
BPH	*Prunus* *domestica*	BPH patients (140)	open-label single-arm study; 100 mg Prosman™ twice daily; no placebo 12 weeks	↓ IPSS; ↓ prostate volume; ↓ PSA	[83]
neuroprotective	*Prunus* *cerasus*	adults (20)	randomized, double-blind, placebo-controlled crossover; 7 days juice concentrate 2 × 30 mL, no placebo; the juice was not analyzed phytochemically	↑ melatonin; ↑ total sleep time & efficiency	[90]
neuroprotective	*Prunus* *cerasus*	older adults (15)	randomized double-blind crossover; cherry juice (237 mL/day; 14 days)	Significant sleep improvement	[89]

Study on human subjects, BPH—benign prostatic hyperplasia; BP—blood pressure; HDL—high-density lipoprotein; IPSS—International Prostate Symptom Score; LDL—low-density lipoprotein; PSA—prostate-specific antigen; ↑ increase; ↓ decrease.

## Data Availability

No new data were created or analyzed in this study.

## Supplementary Materials

The following supporting information can be downloaded at: https://www.mdpi.com/article/10.3390/plants15121891/s1, Table S1. Antioxidant activity of the fruits of *Prunus* species [91,92,93,94,95,96,97].

## Abbreviations

The following abbreviations are used in this manuscript:

CD3+CD4+	Cluster of differentiation 3 and 4 positive T lymphocytes
COX-1	Cyclooxygenase-1
COX-2	Cyclooxygenase-2
CRP	C-reactive protein
fMLP	N-Formylmethionyl-leucyl-phenylalanine
hsCRP	High-sensitivity C-reactive protein
IC_50_	Half maximal inhibitory concentration
ICAM-1	Intercellular adhesion molecule 1
IL-1β	Interleukin 1 beta
IL-6	Interleukin 6
IL-8	Interleukin 8
IL-10	Interleukin 10
iNOS	Inducible nitric oxide synthase
IRAK-1	Interleukin-1 receptor-associated kinase 1
LOX	Lipoxygenase
LPS	Lipopolysaccharide
MAPK	Mitogen-activated protein kinase
miR-146a	MicroRNA-146a
NF-κB	Nuclear factor kappa B
NO	Nitric oxide
Nrf2	Nuclear factor erythroid 2-related factor 2
PGE2	Prostaglandin E2
PMACI	Phorbol 12-myristate 13-acetate and calcium ionophore A23187
RANTES	Regulated upon Activation, Normal T-cell Expressed and Secreted (CCL5)
ROS	Reactive oxygen species
TLR4	Toll-like receptor 4
TNF-α	Tumor necrosis factor alpha
AFP	Alpha-fetoprotein
ALT	Alanine aminotransferase
AST	Aspartate aminotransferase
ATM	Ataxia-telangiectasia mutated kinase
Bax	Bcl-2–associated X protein
Bcl-2	B-cell lymphoma 2 protein
BP	Blood pressure
CaOx	Calcium oxalate
CAT	Catalase
CEA	Carcinoembryonic antigen
CHEK1	Checkpoint kinase 1
CHEK2	Checkpoint kinase 2
GPx	Glutathione peroxidase
GSH	Reduced glutathione
HO-1	Heme oxygenase-1
LDH	Lactate dehydrogenase
MDA	Malondialdehyde
Meth-A	Methylcholanthrene-induced fibrosarcoma cell line
P21	Cyclin-dependent kinase inhibitor 1 (CDKN1A)
PGC-1α	Peroxisome proliferator-activated receptor gamma coactivator 1-alpha
SDA	Seed-derived extract
SOD	Superoxide dismutase
TAC	Total antioxidant capacity
TAG	Triacylglycerols
Telomerase	Telomere-maintaining reverse transcriptase enzyme
XO	Xanthine oxidase
BPH	benign prostatic hyperplasia
HDL	high-density lipoprotein
IPSS	International Prostate Symptom Score
LDL	low-density lipoprotein
PSA	prostate-specific antigen
