# Supplementary material for: Biological Activity and Potential Health Benefits of Edible Prunus Fruits: A Narrative Review
Source: Plants (Basel). 2026 Jun 18;15(12):1891. doi: 10.3390/plants15121891 (PMC13307114; doi:10.3390/plants15121891)
Supplement: Supplementary file 1 [file plants-15-01891-s001.zip › plants-4339176-supplementary.pdf]

Supplementary Information

Article title: Health benefits of the fruits of the edible *Prunus* Species: A Comprehensive Review

Author names: Piotr Służały[1,2], Irma Podolak[2], Agnieszka Galanty[2]

Affiliations: [1]Doctoral School of Medical and Health Sciences, Jagiellonian University Medical College, 16 Łazarza Str., 31-530 Cracow, Poland

[2]Department of Pharmacognosy, Jagiellonian University Medical College, Medyczna 9, 30-688 Kraków, Poland

* **Corresponding author:** agnieszka.galanty@uj.edu.pl

Table S1. Antioxidant activity of the fruits of *Prunus* species.

Species	Plant Material / Extract	Assay	Key Results	Reference
Prunus armeniaca (apricot)	Fresh fruit extract (varieties: Irani, Tilton)	DPPH FRAP	DPPH: IC_{50}: 21.32 ± 11.17 – 92.03 ± 12.02 µg/g FW; Irani (21.32 µg/g FW) highest activity; Tilton (32.03 µg/g FW) FRAP: Irani: 7.62 ± 5.15 mMFe2+eq./g FW; Tilton 6.76 ± 3.09 mMFe2+eq./g FW	[20]
Prunus avium (sweet cherry)	Dried fruit methanol extract	Antioxidant potential (%)	91.16% inhibition	[40]
	Fruit extract encapsuled in naanoparticles	Effect on human emblical vein endothelial cells	↑ permeation of the extract into chitosan, ↑ protection against oxidative stress	[41]
	Fruit methanolic extract	DPPH ABTS FRAP	104.79-288.11µmol Trolox/g DW 172.10 7.01- 364.53±3.07µmol Trolox/g DW FRAP: 147.50±1.51-345.58±9.21 µmol Trolox/g DW	[91]
	17 fruit cultivars	DPPH	29.88%-86.94% of neutralised free radicals	[92]
	Traditional cultivars	DPPH (Trolox equivalent)	128.9 ± 3.38 – 632.3 ± 6.12 mmol TE/100 g FW	[42]
	Commercial cultivars	DPPH (Trolox equivalent)	326.5 ± 79.1 – 692.9 ± 5.88 mmol TE/100 g FW	[42]

Species	Plant Material / Extract	Assay	Key Results	Reference
	Fruit powder of Techlovan cultivar	ABTS<br>FRAP<br>ORAC	25.0 ± 1.3 mmol Trolox/100 g<br>19.5 ± 0.9 mmol TE/100 g<br>117.3 ± 2.6 mmol/100 g	[53]
Prunus cerasoides	—	—	No antioxidant data found	—
Prunus cerasus (sour cherry)	Fully vs. partially ripened fruit	ORAC; DPPH	ORAC: 2.54 ± 0.22 vs. 1.96 ± 0.25 mmol TE/100 g FW; DPPH: 157 ± 18 vs. 115 ± 21 µmol TE/100 g FW	[43]
	Juice (in vivo, mice)	SOD, CAT, GPx	↑ SOD (erythrocytes $P<0.002$; liver $P<0.001$); ↑ GPx (liver $P<0.003$); CAT unchanged	[44]
	21 cultivars of fruit	DPPH	510.62 – 984.81 µmol TE/100 mg	[93]
	Fruit powder of Lutowka cultivar	ABTS<br>FRAP<br>ORAC	36.7 ± 1.5mmol Trolox/100 g<br>21.8 ± 1.8 mmol TE/100 g<br>125.6 ± 1.9mmol/100 g	[53]
Prunus domestica (plum)	Fruit extract	DPPH	IC_{50}: 34.28 ± 2.08 µg/mL	[22]
	Skin vs. flesh	DPPH<br>ORAC	Skin IC_{50}: 45.19±0.12 µg/mL;<br>Flesh IC_{50}:5.95±0.14 µg/mL<br>Skin 14.55±0.21mmol TE/g Extract<br>Flesh 13.02±0.29mmol TE/g Extract	[22]
	Ethyl acetate fraction	Total antioxidant capacity	205.82 ± 2.52 – 1554.15 ± 5.73 µg AAE/mL	[45]
	Flesh and skin of fruit extract	DPPH<br>ORAC	Skin IC_{50}: 4.85±0.04 µg/mL;<br>Flesh IC_{50}:4.39±0.15 µg/Ml<br>Skin 12.55±0.40mmol TE/g<br>Flesh 12.22±0.1740mmol TE/g	[31]
	Karaca, Üryani cultivars	FRAP; DPPH	FRAP: 19.36 ± 8.66 (Karaca), 23.74 ± 7.12 (Üryani) mmol Fe(II)/g; DPPH: 4.19 ± 1.11, 1.03 ± 0.32 mg TE/g	[46]
Prunus mahaleb	Fruit methanolic extract	DPPH	86.25%	[47]
P. padus	fruit	FRAP<br>DPPH	FRAP:45.8 mmol Fe^{2+}/kg;<br>DPPH: 35.8 mmol Trolox/kg	[34]
Prunus persica (peach)	Butanol & hexane fraction	DPPH	IC_{50}: 0.155 mg/mL (butanol); 1.008 mg/mL (hexane)	[45]

Species	Plant Material / Extract	Assay	Key Results	Reference
	Various cultivars (unripe)	FRAP; DPPH; TPC	‘Andross’: FRAP 7.33 mmol AAE/kg; DPPH 5.12 mmol AAE/kg; TPC 1465.32 mg GAE/kg. ‘Everts’: FRAP 8.57; DPPH 6.08; TPC 1714.53 mg GAE/kg	[48]
	Pulp, peel, seed (ethanol extract)	DPPH; ABTS; FRAP	DPPH IC_{50}: pulp 12.0 µg/mL; peel 45.3 µg/mL; FRAP: pulp 30.2; peel 78.9 µM Fe(II)/g	[49]
	Five varieties	DPPH	31.89 ± 0.31 – 728.98 ± 3.74 µM TE/100 g FW; “Filina” highest	[50]
	Fresh vs. preserved pulp (in vivo, rats)	Oxidative stress markers	Fresh pulp & peel > preserved pulp (better cytoprotection)	[51]
	Powder extract of fruit	DPPH ABTS	DPPH: 42.91 mg TE/g; ABTS: 148.21 mg TE/g	[94]
P. serotina	Fruit ethanolic extract	DPPH; Reducing power Redox; potential	73.47 ± 0.01%, 250 µg/mL TE 3.164±0.12 (abs 700nm) 395±2 mV	[95]
P. spinosa	Ethanolic fruit extract	DPPH; ORAC	18.56µmol AAE/g TE 146.09 µmol ascorbic acid equivalent (AAE)/g	[96]
	Methanolic extract of fruit	DPPH ABTS	IC_{50}: 0.0729 ± 0.0348 mg/mL IC_{50}: 0,1896±0,1143 mg/mL	[37]
	Fresh juice of fruit	ORAC DPPH	36.0 µmol Trolox equivalents per gram of fruit 65.25%	[97]

AAE – Ascorbic Acid Equivalent; ABTS – 2,2′-Azino-bis(3-ethylbenzothiazoline-6-sulfonic acid); abs – absorbance; CAT – catalase; DPPH – 2,2-diphenyl-1-picrylhydrazyl; DW – dry weight; eq. – equivalent; Fe(II) – ferrous ion; FRAP – Ferric Reducing Antioxidant Power; FW – fresh weight; GAE – gallic acid equivalent; GPx – glutathione peroxidase; IC_{50} – half maximal inhibitory concentration; ORAC – Oxygen Radical Absorbance Capacity; P – probability value; SOD – superoxide dismutase; TE – Trolox equivalent; TPC – total phenolic content.
